# Estimated Cardiorespiratory Fitness and Metabolic Risks

**DOI:** 10.3390/ijerph21050635

**Published:** 2024-05-16

**Authors:** Robert A. Sloan

**Affiliations:** Division of Social and Behavioral Medicine, Kagoshima University Graduate Medical School, Kagoshima 890-8520, Japan; rsloan@m.kufm.kagoshima-u.ac.jp; Tel.: +81-99-275-5751; Fax: +81-99-275-5749

**Keywords:** cardiorespiratory fitness, estimated CRF, metabolic risk factors, noncommunicable diseases, public health, eCRF prediction models, hypertension, hyperglycemia, dyslipidemia, obesity

## Abstract

This review focuses on the emerging evidence for the association between non-exercise fitness testing, estimated cardiorespiratory fitness (eCRF), and metabolic risk factors. Given the challenges associated with directly measuring cardiorespiratory fitness (CRF) in large populations, eCRF presents a practical alternative for predicting metabolic health risks. A literature search identified seven relevant cohort studies from 2020 to 2024 that investigated the association of eCRF with hypertension, hyperglycemia, dyslipidemia, and obesity. This review consistently demonstrates an inverse relationship between higher eCRF and a lower incidence of metabolic risks, which is in line with CRF cohort studies. It highlights the importance of low eCRF as a primordial indicator for metabolic risks and underscores the potential for broader application. Future research directions should include exploring eCRF’s predictive ability across diverse populations and health outcomes and testing its real-world applicability in healthcare and public health settings.

## 1. Introduction

According to the recent Global Burden of Disease Study, noncommunicable diseases (NCDs) are the primary cause of death, accounting for 74% of all annual mortality [1]. Most of these deaths happen prematurely, before the age of 70. Cardiovascular diseases are the leading cause of mortality related to NCDs, resulting in the loss of 17.9 million lives annually. They are followed by cancer, type 2 diabetes (DM), and kidney disease, predominantly caused by DM [1]. Modifiable health behaviors, such as tobacco use, physical inactivity, and poor diet, significantly contribute to an increase in modifiable metabolic risk factors, including hypertension, hyperglycemia, dyslipidemia, and obesity [2]. The interplay of these risk factors increases the risk of NCD morbidity and mortality. With rising global NCD incidence rates, implementing nuanced approaches targeting metabolic risk factors may help with NCD prevention.

### 1.1. Cardiorespiratory Fitness and Metabolic Health

Vast evidence indicates that low cardiorespiratory fitness is a better prognostic marker of the incidence of morbidity and mortality than inactivity, sedentarism, smoking, overweight, high cholesterol, and high blood pressure [3,4,5,6,7]. Objectively measured cardiorespiratory fitness (CRF) is more comprehensive than traditional risk factors, and according to the American Heart Association, “CRF is directly related to the integrated function of numerous systems, and it is thus considered a reflection of total body health” [6]. CRF measures the ability of an individual’s cardiovascular and respiratory systems to supply oxygen to muscles during aerobic activities, serving as an indicator of mitochondrial function and efficiency. It is directly affected by physical activity, smoking, sedentary behavior, body weight, genetics, age, health status, and biological sex [4,8,9,10]. The gold standard to objectively measure CRF is a laboratory-conducted cardiopulmonary exercise test (CPET) measuring peak oxygen consumption expressed as a relative VO_2_ peak in mL O_2_/kg per minute. A highly correlated (r = 0.96) alternative, maximal graded exercise testing (GXT), is often used in clinical settings for diagnostic purposes [11]. A GXT estimates the VO_2_ peak and is expressed as a peak metabolic equivalent (MET), where 1 MET is equivalent to a resting value of 3.5 mL O_2_/kg per minute [4,6]. In a large meta-analysis of 102,980 healthy adults from baseline, Kodama et al. found that a low peak CRF of <8 METS is a risk factor for all-cause mortality, coronary heart disease, and vascular disease [12]. They also established age- and sex-specific low CRF thresholds per decade (e.g., <9 METS for men and <7 METS for women at age 40, reducing to <8 and <6 METS at age 50, and further to <7 and <5 METS by age 60). In epidemiological investigations, researchers typically categorize low CRF by age and sex into the lowest decile, quartile, or tertile. Notably, America and Japan have established reference standards that specify age- and sex-specific categories for low CRF [13,14].

Recent meta-analyses highlight the importance of objectively measuring CRF to independently predict NCDs related to poor metabolic health in healthy adults from baseline. The persistent finding is the independent inverse association between CRF and NCDs amongst covariates. Low CRF is associated with a higher incidence of developing cardiovascular disease relative risk (95% CI) 1.56 (1.39–1.75), higher CRF has an inverse association with lower DM incidence, hazard ratio (HR) (95% CI) is 0.62 (0.49–0.77), and chronic kidney disease (CKD) incidence 0.58 (0.46–0.73) [12,15,16]. Longitudinal studies collectively show a robust inverse relationship between low CRF and all-cause mortality relative risk (95% CI) 1.70 (1.51 to 1.92), further emphasizing its significance for a healthier and longer lifespan [12]. Despite strong evidence supporting its inclusion, economic and logistical challenges limit the adoption of CRF for routine use in health care and public health.

**Figure 1:**
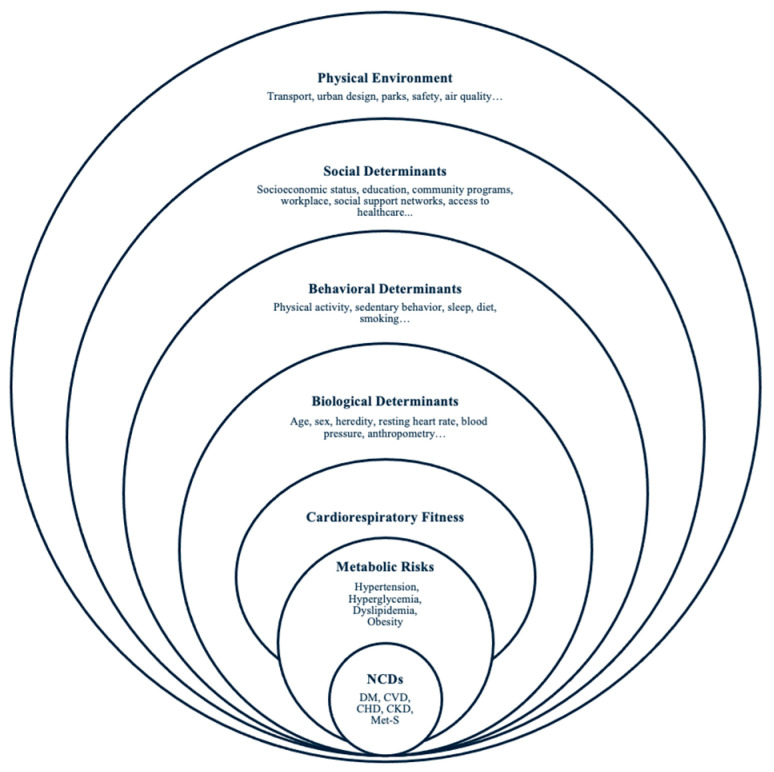
Conceptual framework of the determinants of CRF (adapted from Zeiher et al. [20]).

Using CRF as a prognostic indicator supports a systems-based approach to the primordial and primary prevention of metabolic health risks. An adapted version of a conceptual framework presented by Perumal et al. and Zeiher et al. of the determinants of CRF helps illustrate the interconnectedness of physical, social, behavioral, and biological determinants influencing CRF, metabolic health, and NCDs (Figure 1) [17,18]. The framework is based on social-ecological theory and underscores the need for individual, community, and public health initiatives to improve CRF and reduce metabolic risk factors that lead to NCDs [19,20].

### 1.2. Limitations of Measured CRF in Healthcare and Public Health

CPET and GXT are the most precise methods for objectively measuring CRF to predict health outcomes. However, their practical application faces challenges that hinder their widespread use. These challenges include clinical guidelines, high costs, time requirements, and the necessity for specialized staff and equipment. Such obstacles make routine CRF assessment impractical in healthcare and community settings [21]. These limitations are also apparent when conducting epidemiological investigations on metabolic health outcomes. About eleven unique cohorts, such as the Aerobic Center Longitudinal Study (ACLS), are available for longitudinal analyses, containing healthy adults and measuring CRF at baseline [22].

In response to these limitations, there has been a growing emphasis on developing non-exercise fitness testing equations to estimate CRF (eCRF). These equations use readily available data, such as self-reported physical activity levels, weight, and age, often found in electronic health records or collected through population health surveys. Recent reviews by Ross et al. and Wang et al. of eCRF equations have shown that these models yield moderate (R^2^ = 0.60) to high correlations (R^2^ = 0.80) with directly measured CRF among generally healthy adults [6,21]. Artero et al. conducted a pioneering study in 2014 on the predictability of eCRF concerning all-cause mortality and heart disease among Caucasian Americans, finding that low eCRF predicts health outcomes as effectively as low CRF [23]. However, most equations were developed using samples of Caucasian populations, potentially limiting their applicability across different ethnicities. The 2019 overview by Wang et al. identified that no eCRF studies had been conducted on metabolic health outcomes [21]. Since then, there has been a gradual rise in cohort studies utilizing eCRF to assess the incidence of metabolic health risks.

Given the recent increase in studies since Wang et al.′s 2019 review, the aim of this review is two-fold. First, synthesize the existing longitudinal research on the association between eCRF and metabolic risk factors in adult populations. Second, identify and discuss gaps in the current literature, highlighting areas for future research and practice.

## 2. Literature Search

This review was conducted in PubMed, Scopus, and Web of Science. The search focused on cohort studies that utilized non-exercise fitness tests to estimate CRF and examined the longitudinal relationships between eCRF and metabolic risk factors, including hypertension, hyperglycemia, dyslipidemia, and obesity. Keywords used in the search encompassed combinations of “estimated cardiorespiratory fitness”, “non-exercise fitness test”, “metabolic health risks”, and specific conditions such as “hypertension”, “hyperglycemia”, “dyslipidemia”, and “obesity”. Seven studies were identified for review and published from 2020 until 2024. Table 1 provides a summary of the literature search.

## 3. eCRF and the Incidence of Metabolic Risks

### 3.1. Hypertension

According to the WHO, elevated blood pressure is the primary metabolic risk factor responsible for the highest number of deaths worldwide, accounting for 19% of global mortality [31]. Since 2020, five epidemiological eCRF studies have examined the association of eCRF with the incidence of hypertension [24,25,26,27,30]. Cabanas-Sanchez et al. conducted a large cohort study to examine the long-term relationship (5.7 ± 4.4 years) between eCRF and key metabolic risk factors for adult cardiovascular disease. The study encompassed 200,039 healthy adults (38.5 ± 12.1 years) (50% women) from the Taiwan MJ Cohort (TMJC). The ACLS Jackson eCRF equations were used [9]. The sex-specific Jackson equations include age, body mass index, waist circumference, physical activity index, resting heart rate, and smoking as parameters to calculate eCRF (File S1). From baseline, per 1-MET increase in eCRF was inversely associated with hypertension in middle-aged men and women, respectively (hazard ratio, HR = 0.76, 95% CI, 0.75–0.78 and HR = 0.74, 95% CI, 0.72–0.76) [26]. A sub-analysis also found that minor improvements in eCRF overtime were associated with slightly lower incident rates.

In line with the findings of Cabanas-Sanchez et al., Patel et al. specifically investigated the association between eCRF and hypertension incidence within healthy middle-aged adults (42.8 ± 9.0 years) from the Caucasian population (N = 5513, 20.1% women) from the ACLS cohort [25]. The average follow-up time was five years from baseline. Using the Jackson eCRF equation, the results support the inverse association observed by Cabanas-Sanchez et al. Men in the highest eCRF tertile had an HR = 0.74 (95% CI, 0.68–0.81) compared to those in the lowest tertile. Likewise, the risk reduction for high eCRF was greater for women, HR = 0.64 (95% CI, 0.51–0.81). In addition, a dose-response relationship was found in the cohort. Overall, every 1-MET eCRF increment corresponded to an HR = 0.90 (95% CI, 0.87–0.93) decrease in the incidence of hypertension in the overall cohort [25]. Furthermore, when each parameter of the eCRF equation was also considered, higher-fit, non-smoking, and active individuals had the lowest risk.

Zhao et al. conducted a prospective cohort study on hypertension incidence and dynamic changes over a six-year follow-up period [27]. The study involved 9848 (61% women) late middle-aged adults (51.0 ± 8.5 years) without chronic disease at baseline from the Rural Chinese Cohort Study (RCCS). The main results showed a reduction in hypertension risk of 0.86 (95% CI: 0.84–0.90) per 1 MET increase, aligning with the incremental findings of Cabanas-Sanchez et al. and Patel et al. Furthermore, those who improved their eCRF by ≥2% had a decrease in the incidence of 0.76 (95% CI: 0.59–0.97). Conversely, those with an eCRF decrease of >2% had a higher risk, 1.52 (1.28–1.79).

Lee et al. investigated the association between eCRF in healthy older adults (61.5 ± 9.2 years) and the incidence of cardiometabolic outcomes, including hypertension, across a 15-year follow-up period. The Framingham Offspring (FOS) cohort of 2962 Caucasian participants (52.7% women) was used [24]. Unique to the study was the association of midlife eCRF with hypertension incidence, which was analyzed using three different methods. The first was a single examination of eCRF during the final follow-up period. Second, eCRF trajectories were determined by examining the initial and final periods. Third, risk was determined based on the mean eCRF between examination periods. Low eCRF was defined as the lowest tertile reference or trajectory group. When comparing low eCRF with high single examination eCRF, there was a lower risk of developing hypertension, HR = 0.63 (95% CI, 0.46–0.85). Additionally, high eCRF trajectories and high mean eCRF were associated with a lower risk of hypertension, HR = 0.54 (95% CI, 0.34–0.87) and HR = 0.48 (95% CI, 0.34–0.68), respectively [24]. 

Rather than focusing on incidence, Liu et al. conducted a 4-year investigation utilizing data from the China Health and Retirement Longitudinal Study (CHARLS) to examine eCRF and its impact on changes in an array of metabolic risk factors, including blood pressure. The population included 4862 (52.6% female) older Chinese adults aged 58.6 (9.4) [30]. Their results indicate that in the total population, those with higher baseline eCRF tend to have better arterial pressure, characterized by lower SBP and DBP per year. Those with higher baseline eCRF had significantly (*p* < 0.0001) lower SBP (β, 95% CI; −0.39, −0.52–−0.25) and lower DBP (β, 95% CI; −0.19, −0.28–−0.10) per year [30]. The annual change in eCFR per year was similar for DBP but not SBP.

These five studies provide longitudinal evidence for the independent inverse relationship between eCRF, hypertension, and elevated blood pressure. Higher eCRF was consistently associated with lower hypertension incidence across different populations, age groups, and time frames. The TMJC used the lowest quintile to define low eCRF; the ACLS and FOS cohorts used the lowest tertile; and the RCCS used the lowest quartile. The findings also suggest a dose-response relationship; for every 1-MET increase in eCRF, adults had a 10~25% decrease in risk. While none of the investigations were able to cross-validate eCRF with CRF, the overall findings align with a recent systematic review and meta-analysis on the association of measured CRF and the risk of hypertension, where 1-MET increments in CRF corresponded to an 8% decrease in hypertension in adults [32].

A fundamental similarity across the investigations was that the ACLS Jackson equations were used to determine eCRF. Notably, one of the limitations of the Lee et al. [24]. investigation was that the self-reported physical activity data necessary for the Jackson eCRF calculations were unavailable for some of the examination years. Although the equation was initially validated in a large Caucasian population with mortality as the outcome, the overall findings suggest that low eCRF in generally healthy adults at baseline may serve as a predictor of the onset of hypertension later in life.

### 3.2. Hyperglycemia

The global diabetes prevalence rose from 108 million in 1980 to 537 million in 2021. Projections indicate that this figure will increase to 643 million by 2030 and 783 million by 2045. DM has a substantial impact on global mortality and morbidity, resulting in approximately 6.7 million deaths in 2021, and it increases the likelihood of severe complications such as blindness, kidney failure, heart attacks, stroke, and amputation [33]. The International Diabetes Federation highlights the importance of prevention and early detection in addressing the worldwide spread of DM [34]. Blood tests such as impaired glucose tolerance and fasting glycemia can identify prediabetes and aid in the primary prevention of DM [35]. Recent meta-analyses show that early identification of those with combined low CRF and normal blood glucose may show early signs of insulin resistance and provide prevention opportunities [15,22,36]. From the findings of the meta-analyses, researchers estimated that a 1-MET improvement in CRF leads to clinically significant (5–10%) decreases in the risk of developing DM, impacting public health [15,22,36].

Five cohort studies have explored the relationship between eCRF and hyperglycemia, focusing mainly on the development of DM. As previously described, Lee et al. and Cabanas-Sanchez et al. investigated eCRF and cardiometabolic risks. Lee et al. derived HRs for the onset of DM using three distinct analyses within the FOS cohort. Their findings indicated a significant inverse relationship between eCRF and DM risk; the highest tertile of eCRF was linked to a reduced risk of developing DM (HR = 0.38, 95% CI, 0.23–0.62), with similar protective effects observed across high eCRF trajectories (HR = 0.27, 95% CI, 0.15–0.48) and mean eCRF (HR = 0.31, 95% CI, 0.18–0.54) [24]. Cabanas-Sanchez et al. also found evidence that eCRF can predict the incidence of DM in the TMJC. Two separate analyses investigated the incidence of DM from baseline eCRF, and the other analysis investigated the impact of changes in eCRF over time. Overall, for every 1-MET increase in eCRF, there were corresponding reductions in risk in early middle-aged men and women, respectively. From baseline, men had an HR = 0.67 (95% CI, 0.66–0.69), and women had an HR = 0.64 (95% CI, 0.61–0.66) [26]. When considering changes in eCFR over time, men’s HR = 0.75 (95% CI, 0.69–0.81) and women’s HR = 0.64 (0.57–0.72).

While the TMJC primarily comprised early middle-aged adults of Chinese ethnicity with a higher socioeconomic status, Zhao et al. conducted a comparable study involving 11,825 late middle-aged adults from the RCCS of Chinese ethnicity. The average of a six-year follow-up from baseline was used to determine the association of eCRF with the incidence of DM in men and women. Men in the highest eCRF quartile had an HR = 0.37 (0.22–0.62) compared to the lowest. For every 1-MET increase, there was an HR = 0.69 (0.62–0.78) [28]. Women in the highest eCRF quartile had an HR of 0.56 (0.36–0.88) compared to the lowest. For every 1-MET increase, there was an HR = 0.71 (0.62–0.88). For the total adult population, for every 1-MET increase, there was an HR = 0.89 (0.84–0.95). The four-year CHARLS results supported these inverse associations in the oldest Chinese populations studied. Using the Jackson eCRF as the exposure variable, findings indicate that in the total population, older adults with higher baseline eCRF tend to have significantly (*p* < 0.0001) lower (β, 95% CI; −0.037, −0.05–−0.03) fasting blood glucose per year [30]. This association was consistent with changes in eCRF over time and in men and women, respectively.

Though the ACLS Jackson eCRF type equations are advantageous, integrating them with electronic health records might encounter challenges related to accessibility, primarily because the entry of self-reported physical activity levels is not universally standard in healthcare settings [37]. To overcome this potential barrier, Sloan et al. developed nuanced eCRF equations designed primarily for electronic health records without using physical activity status as an equation parameter [38]. The sex-specific equations were initially validated from the original ACLS cohort (N = 42,676) and compared to the measured CRF for accuracy (File S2). The ACLS Sloan equations incorporate universal parameters that can be derived from electronic health records, including resting heart rate, height, weight, blood pressure, and smoking status. To test the predictability of eCRF with a health outcome, Sloan et al. investigated the incidence of abnormal blood glucose (prediabetes/DM) in 8602 healthy adults from baseline (17.8% women) with a mean age of 43.03 (±8.94) using the ACLS cohort with an average of 5 years of follow-up [29]. Separate analyses were conducted for eCRF and CRF to determine the respective incidence of abnormal blood glucose. A significant inverse relationship was found for both fitness measures. Specifically, for every 1-MET increment, HRs for eCRF and CRF were determined to be 0.96, eCRF (95% CI: 0.93–0.99), and CRF (95% CI: 0.94–0.98), respectively.

Overall, these studies suggest that higher eCRF is independently and inversely associated with the development of hyperglycemia. Evidence from various cohorts, including the ACLS, FOS, TMJC, RCCS, and CHARLS, consistently supports this relationship across different populations and age segments. TMJC and RCCS, both six-year cohort studies focusing on Chinese adults that employed the ACLS Jackson eCRF, demonstrated similar findings. This protective effect was observed regardless of variations in age, health behaviors, socioeconomic status, and environmental conditions. The types of covariates were generally similar across cohorts. Some of the cohorts did not account for prediabetes at baseline, which is a confounder for the increased risk of DM. Though only the Sloan et al. study cross-validated eCRF with CRF, the overall findings from the eCRF studies are generally aligned with CRF meta-analyses [15,22,36]. Notably, from the collective CRF meta-analyses, women accounted for only ~16% of the general population. This underrepresentation of women is inherent in CRF cohort studies due to the lack of CPET and GXT testing data [29,39].

### 3.3. Dyslipidemia

Dyslipidemia includes elevated cholesterol, low-density lipid cholesterol, triglycerides, or reduced high-density lipid cholesterol [26]. Heredity and unhealthy lifestyle health behaviors increase the chance of developing dyslipidemia, which increases the risk for cardiovascular disease. Hypercholesterolemia (elevated low-density lipids), a leading form of dyslipidemia, has escalated as a risk factor for death globally, moving from the 15th position in 1990 to the 8th by 2019, indicating a growing burden of cardiovascular disease risk [40]. Atherogenic dyslipidemia, marked by high triglyceride and low high-density lipid-cholesterol levels, is especially common in individuals with DM, or metabolic syndrome, exacerbating cardiovascular risks [26].

A dearth of research has been conducted on the association between eCRF and dyslipidemia incidence. From the CHARLS cohort, Liu et al. investigated the annual changes in dyslipidemia with eCRF [30]. Their results indicate that older Chinese adults with higher baseline eCRF tend to have better lipid profiles over time. Those with higher baseline eCRF had a significant (*p* < 0.0001) decrease in triglycerides per year of (β, 95% CI; −0.032 mmol·L, −0.04 to −0.03). This effect was observed in males and females. Significant (*p* < 0.0001) positive changes per year in high-density lipoprotein also occurred (β, 95% CI; 0.005 mmol·L, 0.002 to 0.007). Increases were more significant in males than females.

The findings from the TMJC provided outcomes for hypercholesterolemia and atherogenic dyslipidemia [26]. Separate analyses investigated the incidence of each outcome from baseline eCRF, and the other analyses investigated the impact of changes in eCRF per 1-MET increase over time. From baseline, men had a reduced HR = 0.95 (95% CI, 0.93–0.96), and women had an HR = 0.98 (95% CI, 0.96–1.01) for hypercholesterolemia. When considering changes over time, the findings were similar. For atherogenic dyslipidemia, from baseline, men had an HR = 0.82 (95% CI, 0.80–0.83), and women had an HR = 0.80 (95% CI, 0.78–0.83). When considering per 1-MET changes in eCFR over time, the findings were again similar to those of the baseline analyses.

Collectively, these two investigations in middle-aged and older adults of Chinese ethnicity provide evidence that higher eCFR predicts dyslipidemia. Again, the ACLS Jackson equation was successfully used. The comparability of investigations regarding measured CRF and dyslipidemia is limited. In an ACLS cohort of healthy men (11,418) at baseline, higher CRF was inversely associated with low-density lipid cholesterol and positively associated with HDL. When age was factored in, trajectories revealed that higher CRF in young to middle-aged men delayed abnormal low-density lipid cholesterol by 15 years [41]. Breneman et al. also conducted a study using the ACLS cohort of 9651 patients (15% female). They found that a higher baseline CRF and maintaining fitness (~9 years) were associated with a lower likelihood of atherogenic dyslipidemia [42].

### 3.4. Obesity

The World Health Organization released a 2022 report that obesity rates have doubled since 1990 to 12.2% in men and 15.7% in women, and globally, 1 billion people have obesity [43]. Globally, obesity is responsible for ~5 million deaths annually and is defined as having a BMI ≥ 30 [43]. From a metabolic risk standpoint, a more critical measure of obesity and NCD risk is central fatness, typically measured by waist circumference, with differing cut points set according to biological sex and ethnicity [44]. The health consequences of obesity include an increased incidence of NCDs and premature mortality. Notably, Areto et al. found that eCRF and CRF were superior predictors of all-cause mortality, CVD mortality, and CVD morbidity compared to BMI, or waist circumference, in the ACLS cohort [23]. Concomitantly, a recent CHARLS cohort investigation showed clear dose-response relationships with progressively higher eCRF, predicting a lower incidence of CVD, heart disease, and stroke [45].

No studies to date have investigated the longitudinal relationship of eCRF with the onset of obesity, and very few CRF studies have investigated this relationship. Ortega et al. conducted two retrospective cohort analyses and found that low CRF is associated with a significantly increased risk of abdominal obesity and a BMI ≥ 30 after two years in Spanish adults [46,47]. The limited literature may be partly due to the limited number of cohorts that have measured CRF to conduct this longitudinal analysis. Therefore, eCRF studies using large electronic health records or population data sets may provide a method for further investigation.

## 4. Discussion

Studies on non-exercise fitness testing, eCRF, and metabolic health risks are beginning to emerge in the peer-reviewed literature. To date, seven cohort investigations have been published, providing evidence for the incidence of hypertension, hyperglycemia, and dyslipidemia. No studies have been conducted on eCRF and the incidence of obesity. This review provides emerging evidence for using eCRF as a prognostic indicator for metabolic health risk. Significant inverse and dose-response associations were repeatedly demonstrated between higher eCRF and a lower risk of high blood pressure, blood sugar, and abnormal lipids. These findings are aligned with previous studies using measured CRF. Most CRF cohort studies have been limited to primarily male Caucasian populations [12,15]. However, the increased use of eCRF in population health data sets has begun to expand the evidence on age groups, females, ethnicities, and socioeconomic status.

The limitations identified across some of the eCRF cohort studies in this review include concerns about low sample size, measurement accuracy, confounders, covariates, and generalizability of findings. In 2019, Wang et al. provided a scoping review of more than twenty eCRF equations [21]. At the time, five health eCRF outcome studies focused on mortality as the primary outcome. Since then, the literature has expanded to include metabolic health risk outcomes, as discussed in this review. The ACLS Jackson equation was most commonly used to calculate eCRF in six investigations [9]. The Jackson equation uses self-reported physical activity as one of the equation parameters, initially validated using the ACLS physical activity index [48]. Only the investigation by Patel et al. used the ACLS-validated scale [25]. The FOS, TMJC, RCCS, and CHARLS studies used unvalidated domestically designed questionnaires and adapted the parameter into the equation. This adaptation method likely resulted in misclassification of eCRF levels in some participants, thereby reducing the accuracy and reliability of findings. It is also important to point out that the self-reported physical activity status is prone to bias, leading to misclassification.

Other commonly cited issues are the homogeneous populations studied, often with high socioeconomic status or specific ethnic backgrounds, limiting the external validity of the results. The Wang et al. review also recommended choosing equations that share the same ethnicity and age group. While there are validated eCRF equations for people of Chinese ethnicity, the CHARLS, TMJC, and RCCS used the Caucasian-validated Jackson equation with promising findings aligned with CRF meta-analyses. Notably, most of the participants in the meta-analyses are Caucasian males [22,36].

Another caution when applying eCRF equations is using redundant covariates or confounders in multivariate analyses. For example, when BMI is a parameter in an eCRF equation and is used again as a covariate during analysis, it could lead to multicollinearity. Multicollinearity occurs when two or more predictor variables in a regression model are highly correlated, meaning that one can be linearly predicted from the others with a substantial degree of accuracy [49]. This redundancy may inflate the variance of the coefficient estimates and the standard errors, making statistical tests less reliable, the model’s predictions less precise, and leading to wider confidence intervals. Potential solutions include conducting variance inflation factor (VIF) analysis or transforming a continuous variable by categorizing the covariate or confounder (e.g., 1 = BMI < 30, 2 = BMI ≥ 30) [50]. More recently, the advancement of causal inference through causal machine learning may offer a solution for more accurately accounting for covariates and confounders. Unlike associative studies that incorporate confounding variables to enhance the accuracy of outcome predictions, causal machine-learning models meticulously seek to isolate and exclude the influence of these variables to assess the impact of the exposure variable directly [51]. Furthermore, machine learning methods may be more beneficial when using large amounts of real-world data, such as electronic health records.

With the current dearth of literature, there are ample opportunities for further study regarding eCRF, metabolic risks, NCDs, and a broad range of health outcomes. Potential areas for future research may include focusing on larger multiethnic cohorts and young adults and comparing other eCRF equations for their predictive capability. Also, more evidence across diverse ethnicities of women is needed. One drawback is that a limited number of population health data sets or electronic health records contain CRF or all the parameters (e.g., Jackson) needed to calculate eCRF [38]. Different eCRF equations may need to be applied to access more extensive, heterogeneous cohorts over longer durations. As discussed by Wang et al., eCRF models that do not use self-reported physical activity as a parameter may be applied more broadly (e.g., electronic health records) [21,38].

From a metabolic health outcomes perspective, there are various potential cohort studies to consider. eCRF prediction of prehypertension, prediabetes, and borderline dyslipidemia would be helpful to inform primordial prevention initiatives. Studies focused on the incidence of obesity, metabolic syndrome, and NCDs would add significant value to the growing eCRF prediction literature. Prevalence studies for understanding the fitness level of a particular community, region, or company can help map the magnitude and distribution of low fitness and assist with public health planning. Lastly, conducting experimental intervention studies using changes in eCRF would provide more evidence for the tool’s validity.

A growing and essential area of eCRF research and primary care is net reclassification improvement (NRI) for risk estimation. NRI is a statistical approach that assesses the extent to which incorporating a new biomarker, such as eCRF, improves the classification accuracy of individuals into more appropriate risk categories [5]. For example, physicians often use the Framingham Risk Score to make patient clinical decisions. To improve the accuracy of the 10-year CHD risk score, Gander et al. applied the Jackson eCRF [52]. The study showed that adding the eCRF improves the overall accuracy of the Framingham Risk Score in Caucasian men for heart disease risk. Similar findings were also found in a nationally representative sample of Koreans and a southern Chinese population for CVD mortality and morbidity, respectively [53,54]. There are numerous other risk prediction tools (e.g., DM, CKD, dementia) where adding eCRF may add predictive value.

### Future Directions

CRF has been stipulated as a vital sign by the American Heart Association, and eCRF has been proposed to be used regularly in primary care settings to identify patients with low fitness and provide brief counseling [6,55]. However, a recent meta-analysis and systematic review concluded that the effectiveness of this individualistic approach might not, on its own, improve physical activity, a key determinant of fitness, to sustain itself beyond 6 to 12 months [56,57]. In agreement with this observation, the International Society for Physical Activity and Health states, “Searching for a single solution to increasing physical activity may have hampered progress in this field by encouraging focus on simple, often short-term, individual-level health outcomes rather than complex, multiple, upstream, population-level actions and outcomes [58].” Brief counseling may be more effective when combined with the determinants of eCRF in an individual’s environment [58]. This method is in line with the conceptual framework of the determinants of CRF (Figure 1) and stems from social ecological theory [18]. Consequently, research has to be conducted on using this framework with eCRF.

Given the growing sophistication of technology, eCRF has the potential to be utilized as a population health vital sign to help prevent metabolic and other health risks. Electronic health records can auto-populate eCRF for rapid access and review [29]. Integrating geographic information systems with electronic health record-derived eCRF data can enhance the early identification and mapping of metabolic risk factors [59]. This integration allows public health officials to visually pinpoint areas of low fitness, referred to as hot spots, and further leverage eCRF parameters to segment and target specific populations, such as unfit middle-aged male smokers. Machine learning and artificial intelligence can further augment this process, enabling sophisticated, actionable analyses to guide targeted interventions and potentially maximize the impact of individual, community, and public health initiatives [60].

The use of eCRF in healthcare and public health settings aligns with the International Society for Physical Activity and Health’s Eight Investments That Work for Physical Activity [58]. Both initiatives focus on accessibility to diverse physical activity, exercise, and sports opportunities, facilitating the implementation of strategies like active travel and urban design. Effective collaboration between healthcare systems and public health is crucial for navigating the complex social ecology of communities. Such partnerships are instrumental in planning and deploying a systems-based approach to reduce metabolic risks.

## 5. Conclusions

This review underscores the emerging evidence of eCRF as a primordial indicator of metabolic health risks. The current literature affirms a consistent inverse association between higher eCRF and reduced metabolic risks, highlighting eCRF’s predictive ability to be concomitant with CRF. Future research should aim to explore eCRF’s predictive capability across a broader spectrum of populations and outcomes and evaluate its real-world utility in healthcare and public health settings.

## Figures and Tables

**Table 1:** A summary table of included cohort studies.

First Author, Year of Publication	Mean Follow-Up Years from Baseline (±SD)	Cohort	Location and Sample Size	Sex	Mean Age (±SD)	eCRF Model	Metabolic Risk Outcomes
Lee et al., 2021 [24]	15	Framingham Offspring Study (FOS)	America 2962	M and F	66.2 (8.6)	Jackson	Incidence of SBP ≥ 140/DBP ≥ 90 mm Hg, Incidence of DM fasting glucose level of 126 mg/dL or higher, nonfasting glucose level of 200 mg/dL or higher, or the use of hypoglycemic medications.
Patel et al., 2022 [25]	5	Aerobics Center Longitudinal Study (ACLS)	America 5513	M and F	42.8 (9.0)	Jackson	Incidence of resting SBP ≥ 130/DBP ≥ 80 mm Hg or self-reported, physician-diagnosed hypertension.
Cabanas-Sánchez et al., 2020 [26]	5.7 (4.4)	Taiwan MJ Cohort (TMJC)	Taiwan 200,039	M and F	38.5 (12.1)	Jackson	Incidence of SBP ≥ 140/DBP ≥ 90 mm Hg, serum total cholesterol ≥240 mg/dL, and fasting blood glucose ≥126 mg/dL. Atherogenic dyslipidemia was defined as triglycerides ≥150 mg/dL and HDL-C < 40 mg/dL in men and <50 mg/dL in women.
Zhao et al., 2022 2024 [27,28]	6.01 (Median)	Rural Chinese Cohort Study (RCCS)	China 11,825 9848	M and F	51.0 (8.5)	Jackson	Incidence of DM was defined as fasting plasma glucose 7.0 mmol/L or current treatment with anti-diabetes medication or a self-reported history of DM, gestational diabetes mellitus, or diabetes due to other causes. Incidence of SBP ≥ 140/DBP ≥ 90 mm Hg or antihypertensive medication.
Sloan et al., 2023 [29]	4.87 (4.58)	Aerobics Center Longitudinal Study (ACLS)	America 8602	M and F	43.0 (8.9)	Sloan	Incidence of prediabetes (impaired fasting glucose) or DM as fasting plasma glucose concentrations of 100 to 125 and ≥126 mg/dL, respectively. Those who self-reported DM or hypoglycemic medication during a follow-up were also classified as having abnormal glucose.
Liu et al., 2024 [30]	4 (Median)	China Health and Retirement Longitudinal Study (CHARLS)	China 4862	M and F	58.6 (9.4)	Jackson	Change in resting SBP, DBP, fasting triglycerides, high-density lipoprotein, total cholesterol

SBP = systolic blood pressure, DBP = diastolic blood pressure, DM = Type 2 diabetes, HDL-C = high-density lipoprotein.

## Data Availability

Not applicable.

## Supplementary Materials

The following supporting information can be downloaded at: https://www.mdpi.com/article/10.3390/ijerph21050635/s1, File S1 Jackson et al. [9], and File S2 Sloan et al. [38], equations.
