# Supplementary material for: Estimated Cardiorespiratory Fitness and Metabolic Risks
Source: Int J Environ Res Public Health. 2024 May 16;21(5):635. doi: 10.3390/ijerph21050635 (PMC11120962; doi:10.3390/ijerph21050635)
Supplement: Supplementary file 1 [file ijerph-21-00635-s001.zip › File S1.pdf]

Supplement S1

Jackson et al., equations:

Male:

(METs) = 21.2870 + (0.1654 x age) (0.0023 x age^2) (0.2318 x BMI) (0.0337 x waist circumference) (0.0390 x resting heart rate) + (0.6351 x PAI) (0.4263 x current smoker)

Female:

(METs) =14.7873 + (0.1159 x age) (0.0017 x age^2) (0.1534 x BMI) (0.0085 x waist circumference) (0.0364 x resting heart rate) + (0.5987 x PAI) (0.2994 x current smoker)

In the original equation, Physical activity was assessed with a 5-level physical activity index (PAI) based on a formerly validated questionnaire. Physically active was defined as walking or jogging 10 miles or more per week that was equal to the physical activity index levels 3 and 4; inactive was defined as walking or jogging <10 miles per week, participating in some other regular physical activity such as bicycling, swimming, racquet sports, and other strenuous sports, but not walking or jogging, or no regular activity that was equal to the physical activity index levels 0 to 2.
