# Supplementary material for: Estimated Cardiorespiratory Fitness and Metabolic Risks
Source: Int J Environ Res Public Health. 2024 May 16;21(5):635. doi: 10.3390/ijerph21050635 (PMC11120962; doi:10.3390/ijerph21050635)
Supplement: Supplementary file 1 [file ijerph-21-00635-s001.zip › File S2.pdf]

Sloan et al. equation:

Male

$$
\begin{aligned}
\text{METs} = &-0.70\left(\frac{Age-44.14}{9.49}\right)+0.04\left(\frac{Ht-179.15}{6.51}\right)-0.85\left(\frac{rHR-60.10}{10.45}\right) \\
&-0.96\left(\frac{BMI-26.46}{3.50}\right)+0.24\left(\frac{SBP-120.58}{12.70}\right)-0.08\left(\frac{DBP-81.09}{9.32}\right) \\
&-0.14\left(\frac{Age-44.14}{9.49}\right)^2-0.05\left(\frac{Age-44.14}{9.49}\right)\left(\frac{Ht-179.15}{6.51}\right) \\
&+0.02\left(\frac{Age-44.14}{9.49}\right)\left(\frac{rHR-60.10}{10.45}\right) \\
&+0.05\left(\frac{Age-44.14}{9.49}\right)\left(\frac{BMI-26.46}{3.50}\right) \\
&-0.09\left(\frac{Age-44.14}{9.49}\right)\left(\frac{SBP-120.58}{12.70}\right) \\
&+0.04\left(\frac{Age-44.14}{9.49}\right)\left(\frac{DBP-81.09}{9.32}\right) \\
&+0.07\left(\frac{Wt-85.04}{12.84}\right)\left(\frac{Ht-179.15}{6.51}\right)-0.07\left(\frac{Ht-179.15}{6.51}\right)^2 \\
&+0.04\left(\frac{Ht-179.15}{6.51}\right)\left(\frac{rHR-60.10}{10.45}\right) \\
&-0.05\left(\frac{Ht-179.15}{6.51}\right)\left(\frac{BMI-26.46}{3.50}\right) \\
&-0.01\left(\frac{Ht-179.15}{6.51}\right)\left(\frac{SBP-120.58}{12.70}\right)+0.11\left(\frac{rHR-60.10}{10.45}\right)^2 \\
&+0.13\left(\frac{rHR-60.10}{10.45}\right)\left(\frac{BMI-26.46}{3.50}\right) \\
&+0.03\left(\frac{rHR-60.10}{10.45}\right)\left(\frac{SBP-120.58}{12.70}\right) \\
&-0.01\left(\frac{rHR-60.10}{10.45}\right)\left(\frac{DBP-81.09}{9.32}\right) \\
&-0.09\left(\frac{BMI-26.46}{3.50}\right)\left(\frac{SBP-120.58}{12.70}\right) \\
&+0.04\left(\frac{BMI-26.46}{3.50}\right)\left(\frac{DBP-81.09}{9.32}\right)+0.02\left(\frac{SBP-120.58}{12.70}\right)^2 \\
&-0.06\left(\frac{SBP-120.58}{12.70}\right)\left(\frac{DBP-81.09}{9.32}\right)+0.01\left(\frac{DBP-81.09}{9.32}\right)^2 \\
&+0.88(1-Smoking)+11.29
\end{aligned}
$$

Female

$$
\begin{aligned}
METs = {} & -0.71\left(\frac{Age-44.11}{10.17}\right)+0.09\left(\frac{Ht-164.62}{5.96}\right)-0.63\left(\frac{rHR-64.02}{10.11}\right) \\
& -0.75\left(\frac{BMI-23.31}{3.68}\right)+0.17\left(\frac{SBP-112.42}{13.94}\right)-0.10\left(\frac{Age-44.11}{10.17}\right)^2 \\
& -0.07\left(\frac{Age-44.11}{10.17}\right)\left(\frac{Ht-164.62}{5.96}\right) \\
& +0.08\left(\frac{Age-44.11}{10.17}\right)\left(\frac{rHR-64.02}{10.11}\right) \\
& +0.10\left(\frac{Age-44.11}{10.17}\right)\left(\frac{BMI-23.31}{3.68}\right) \\
& -0.05\left(\frac{Age-44.11}{10.17}\right)\left(\frac{SBP-112.42}{13.94}\right) \\
& +0.02\left(\frac{Wt-63.21}{10.78}\right)\left(\frac{rHR-64.02}{10.11}\right)+0.02\left(\frac{Ht-164.62}{5.96}\right)^2 \\
& -0.01\left(\frac{Ht-164.62}{5.96}\right)\left(\frac{BMI-23.31}{3.68}\right) \\
& +0.07\left(\frac{Ht-164.62}{5.96}\right)\left(\frac{SBP-112.42}{13.94}\right)+0.10\left(\frac{rHR-64.02}{10.11}\right)^2 \\
& +0.05\left(\frac{rHR-64.02}{10.11}\right)\left(\frac{BMI-23.31}{3.68}\right) \\
& -0.02\left(\frac{rHR-64.02}{10.11}\right)\left(\frac{DBP-76.04}{9.33}\right)+0.01\left(\frac{BMI-23.31}{3.68}\right)^2 \\
& -0.06\left(\frac{BMI-23.31}{3.68}\right)\left(\frac{DBP-76.04}{9.33}\right) \\
& -0.03\left(\frac{SBP-112.42}{13.94}\right)\left(\frac{DBP-76.04}{9.33}\right)-0.01\left(\frac{DBP-76.04}{9.33}\right)^2 \\
& +0.77(1-Smoking)+9.07
\end{aligned}
$$

Google sheet link (simplified calculation tool transferable to Excel):
https://docs.google.com/spreadsheets/d/1lnYl4u62AMSUxkzyzKsKBHe8vr3WzLVRYxGyYE7xoGQ/edit#gid=155 8519083
